# Supplementary material for: Comparative genomics provides new insights into the diversity, physiology, and sexuality of the only industrially exploited tremellomycete: Phaffia rhodozyma
Source: BMC Genomics. 2016 Nov 9;17:901. doi: 10.1186/s12864-016-3244-7 (PMC5103461; doi:10.1186/s12864-016-3244-7)
Supplement: Additional file 6: — List of orphan genes with links to PFAM (related to Additional file 1: Table S1). (ZIP 1428 kb) [file 12864_2016_3244_MOESM6_ESM.zip › BLAST_HTML_FTR/G03833_P.html]

BLAST Search Results


```
BLASTP 2.2.27+


Reference:
Stephen F. Altschul, Thomas L. Madden, Alejandro A. Schäffer,
Jinghui Zhang, Zheng Zhang, Webb Miller, and David J. Lipman (1997),
"Gapped BLAST and PSI-BLAST: a new generation of protein database
search programs", Nucleic Acids Res. 25:3389-3402.


Reference for
composition-based statistics:
Alejandro A. Schäffer, L. Aravind, Thomas L. Madden, Sergei
Shavirin, John L. Spouge, Yuri I. Wolf, Eugene V. Koonin, and
Stephen F. Altschul (2001), "Improving the accuracy of PSI-BLAST
protein database searches with composition-based statistics and
other refinements", Nucleic Acids Res. 29:2994-3005.


Database: nr
           71,551,133 sequences; 26,053,659,533 total letters


Query= G03833_P

Length=147
                                                                      Score     E
Sequences producing significant alignments:                          (Bits)  Value

emb|CDZ96306.1|  hypothetical protein [Xanthophyllomyces dendrorh...   286    1e-94
ref|XP_011502667.1|  PREDICTED: spastin isoform X3 [Ceratosolen s...  37.4    4.2  
ref|WP_047997574.1|  BCCT transporter [Puniceibacterium sp. IMCC2...  37.0    5.6  
ref|XP_011502666.1|  PREDICTED: spastin isoform X2 [Ceratosolen s...  37.0    5.8  
ref|XP_011502665.1|  PREDICTED: spastin isoform X1 [Ceratosolen s...  37.0    5.9  
ref|WP_021100363.1|  choline uptake protein BetT [Litoreibacter a...  36.2    8.4  


 >emb|CDZ96306.1| hypothetical protein [Xanthophyllomyces dendrorhous]
Length=267

 Score =  286 bits (732),  Expect = 1e-94, Method: Compositional matrix adjust.
 Identities = 141/142 (99%), Positives = 142/142 (100%), Gaps = 0/142 (0%)

Query  1    MLSQVLPTIVSSIIRSFQSLSSPDSRLLPSNPALSLSVDSHSQLLPQFASFHLVPFFNLV  60
            MLSQVLPTIVSSIIRSFQSLSSPDSRLLPSNPALSLSVDSHSQLLPQFASFHLVPFFNLV
Sbjct  1    MLSQVLPTIVSSIIRSFQSLSSPDSRLLPSNPALSLSVDSHSQLLPQFASFHLVPFFNLV  60

Query  61   LTVVVFLYLIDEQTHADISRCVDTLTGNKYNCSVDIERDPSYVTNEKGLPVYMTPSNASS  120
            LTVVVFLYLIDEQTHADISRCVDTLTGNKYNCSVDIERDPSYVTNEKGLPVYMTPSNASS
Sbjct  61   LTVVVFLYLIDEQTHADISRCVDTLTGNKYNCSVDIERDPSYVTNEKGLPVYMTPSNASS  120

Query  121  TARMSDKEQAVLTGLVAGCLLT  142
            TARMSDKEQAVLTGLVAGCLL+
Sbjct  121  TARMSDKEQAVLTGLVAGCLLS  142


>ref|XP_011502667.1| PREDICTED: spastin isoform X3 [Ceratosolen solmsi marchali]
Length=626

 Score = 37.4 bits (85),  Expect = 4.2, Method: Compositional matrix adjust.
 Identities = 21/57 (37%), Positives = 30/57 (53%), Gaps = 0/57 (0%)

Query  50   SFHLVPFFNLVLTVVVFLYLIDEQTHADISRCVDTLTGNKYNCSVDIERDPSYVTNE  106
            SF L+  FN + TV+  LY+I +  +A  SR +     NK  C ++I  D   V NE
Sbjct  70   SFPLIILFNALRTVIYQLYVIFKYIYASTSRLMQRKHSNKNTCQLEIVVDREAVCNE  126


>ref|WP_047997574.1| BCCT transporter [Puniceibacterium sp. IMCC21224]
 gb|KMK65139.1| choline/carnitine/betaine transport [Puniceibacterium sp. IMCC21224]
Length=537

 Score = 37.0 bits (84),  Expect = 5.6, Method: Composition-based stats.
 Identities = 27/79 (34%), Positives = 43/79 (54%), Gaps = 7/79 (9%)

Query  18   QSLSSPDSRLLPSNPALSLSVDSHSQLLPQFASFHLVPFFNLVLTVVVFLYLIDEQTHAD  77
            Q L+SP++ L+ +N      +DS+S  L  F   + +PF  +  T+ +FL L+   T +D
Sbjct  407  QILTSPETSLVKAN-----VIDSYSPELSLFGMLNGLPFTKIASTIAIFLALVFFVTSSD  461

Query  78   I-SRCVDTLT-GNKYNCSV  94
              S  VDT+T G K +  V
Sbjct  462  SGSLVVDTITAGGKIDAPV  480


>ref|XP_011502666.1| PREDICTED: spastin isoform X2 [Ceratosolen solmsi marchali]
Length=700

 Score = 37.0 bits (84),  Expect = 5.8, Method: Compositional matrix adjust.
 Identities = 21/57 (37%), Positives = 30/57 (53%), Gaps = 0/57 (0%)

Query  50   SFHLVPFFNLVLTVVVFLYLIDEQTHADISRCVDTLTGNKYNCSVDIERDPSYVTNE  106
            SF L+  FN + TV+  LY+I +  +A  SR +     NK  C ++I  D   V NE
Sbjct  70   SFPLIILFNALRTVIYQLYVIFKYIYASTSRLMQRKHSNKNTCQLEIVVDREAVCNE  126


>ref|XP_011502665.1| PREDICTED: spastin isoform X1 [Ceratosolen solmsi marchali]
Length=723

 Score = 37.0 bits (84),  Expect = 5.9, Method: Compositional matrix adjust.
 Identities = 21/57 (37%), Positives = 30/57 (53%), Gaps = 0/57 (0%)

Query  50   SFHLVPFFNLVLTVVVFLYLIDEQTHADISRCVDTLTGNKYNCSVDIERDPSYVTNE  106
            SF L+  FN + TV+  LY+I +  +A  SR +     NK  C ++I  D   V NE
Sbjct  70   SFPLIILFNALRTVIYQLYVIFKYIYASTSRLMQRKHSNKNTCQLEIVVDREAVCNE  126


>ref|WP_021100363.1| choline uptake protein BetT [Litoreibacter arenae]
 gb|EPX78983.1| High-affinity choline uptake protein BetT [Litoreibacter arenae 
DSM 19593]
Length=537

 Score = 36.2 bits (82),  Expect = 8.4, Method: Composition-based stats.
 Identities = 26/79 (33%), Positives = 43/79 (54%), Gaps = 7/79 (9%)

Query  18   QSLSSPDSRLLPSNPALSLSVDSHSQLLPQFASFHLVPFFNLVLTVVVFLYLIDEQTHAD  77
            Q L+SP++ ++ +N      +DS+S  L  F   + +PF  +  T+ +FL L+   T +D
Sbjct  407  QILTSPETSMVKAN-----VIDSYSPELSLFGMLNELPFTKIASTIAIFLALVFFVTSSD  461

Query  78   I-SRCVDTLT-GNKYNCSV  94
              S  VDT+T G K +  V
Sbjct  462  SGSLVVDTITAGGKIDAPV  480


Lambda      K        H        a         alpha
   0.323    0.134    0.388    0.792     4.96 

Gapped
Lambda      K        H        a         alpha    sigma
   0.267   0.0410    0.140     1.90     42.6     43.6 

Effective search space used: 652013415720


  Database: nr
    Posted date:  Sep 23, 2015 12:05 AM
  Number of letters in database: 26,053,659,533
  Number of sequences in database:  71,551,133


Matrix: BLOSUM62
Gap Penalties: Existence: 11, Extension: 1
Neighboring words threshold: 11
Window for multiple hits: 40
```
